# Congenital Aphakia Associated With a *GJA8* Pathogenic Variant: A Case Report

**DOI:** 10.1002/ccr3.70286

**Published:** 2025-05-05

**Authors:** Sarah A. M. Lucas, Elena Franco, Hannah L. Scanga, Nathan L. Clark, Ken K. Nischal

**Affiliations:** ^1^ Department of Human Genetics University of Utah Salt Lake City Utah USA; ^2^ Division of Pediatric Ophthalmology and Strabismus UPMC Children's Hospital of Pittsburgh Pittsburgh Pennsylvania USA; ^3^ Department of Biological Sciences University of Pittsburgh Pittsburgh Pennsylvania USA

**Keywords:** congenital aphakia, congenital corneal opacities, genetic corneal disease, *GJA8*, ocular genetics

## Abstract

Congenital aphakia is a rare eye condition in which the lens fails to form properly. It is typically caused by pathogenic variants within the *FOXE3* or *HCCS* genes; however, it can also be associated with *GJA8* pathogenic variants. *GJA8* should be included in the genetic testing of patients with this condition.

## Graphical abstract

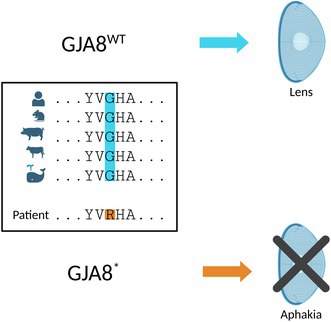

## 1 Introduction

The term “congenital aphakia” (CA) refers to a rare form of anterior segment dysgenesis, where the lens fails to form properly [1]; it can be primary if the lens does not form at all, or secondary when it initially forms but disappears thereafter [2]. The absence of the lens can lead to severe malformations of the anterior segment, including congenital corneal opacity (CCO), iris hypoplasia or aniridia, and glaucoma. Homozygous or compound heterozygous pathogenic variants of the *FOXE3* gene (MIM *601094) are the most frequent known cause of CA; in these cases, CA is usually not associated with extra‐ocular features [3]. Other genetic causes of CA include *HCCS* (MIM *300056) pathogenic variants in the context of microphthalmia linear skin defects syndrome (MLS), but in these cases, extraocular features are present [2, 4, 5].

A recent study [6] identified a *GJA8* missense variant (c.280G>A, p.Gly94Arg) in a male presenting with bilateral CA, corneal opacity, bilateral microphthalmia, iris and optic disc coloboma, and bilateral primary glaucoma. No extraocular anomalies were observed. We herein report another case of bilateral CA associated with a de novo *GJA8* pathogenic variant.

## 2 Case History/Examination

**FIGURE 1:**
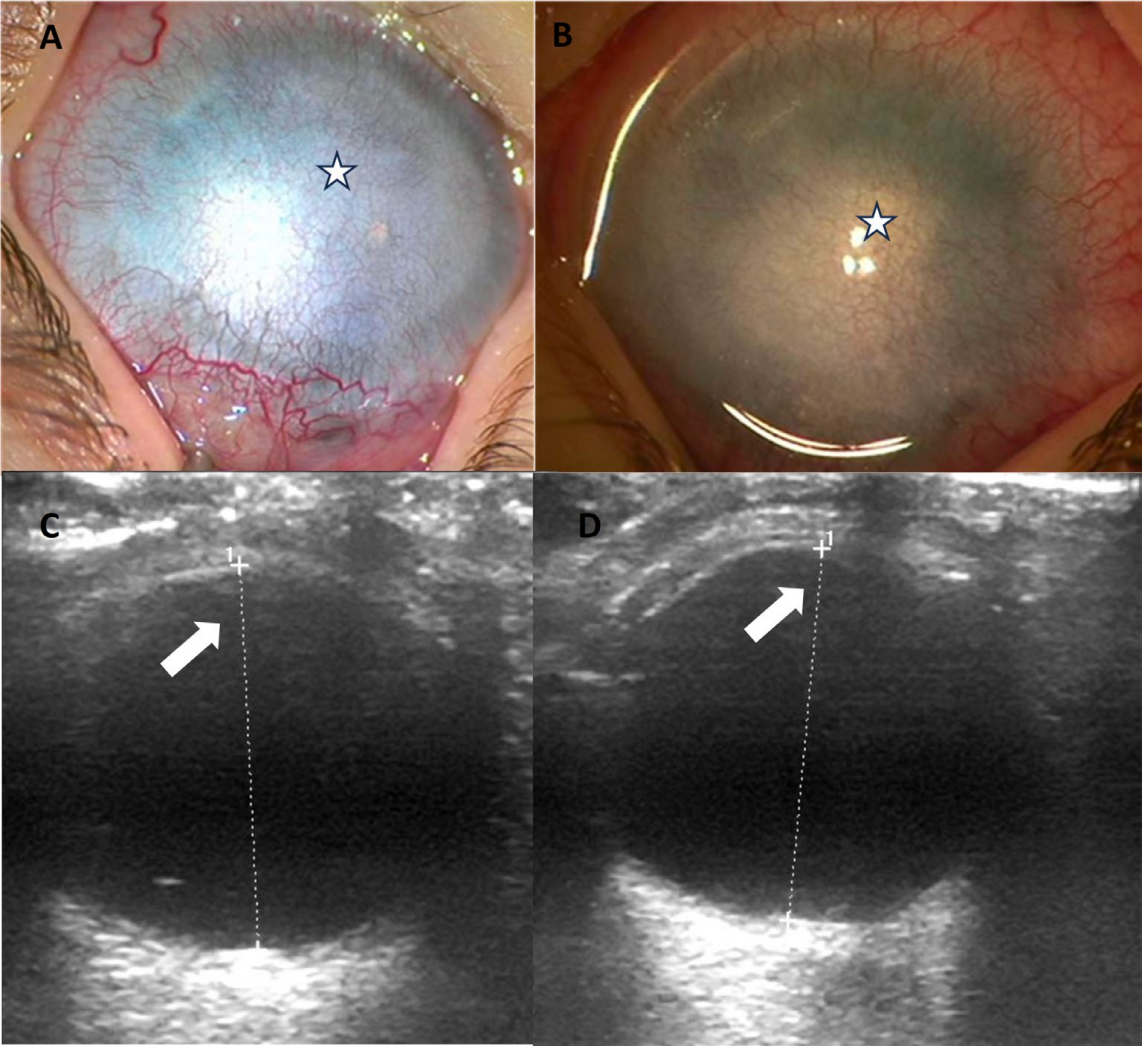
Composite image of right and left eyes with corresponding linear B‐scan ultrasound of the patient. A and B show the silver appearance of the cornea previously described in congenital primary aphakia (white star) with marked vascularization of the cornea. C and D show the linear B‐scan ultrasound which allows visualization of the lens in a normal eye fails to demonstrate the presence of a lens in either eye (white arrows). The axial lengths (dotted white lines) were 15.1 mm in the right eye and 15.2 mm in the left eye, which is less than the normal axial length at 3 months of age (16.5 mm) when this test was performed.

A 2‐week‐old male with a history of bilateral corneal clouding and anterior segment dysgenesis was referred to the Division of Pediatric Ophthalmology, Strabismus, and Adult Motility at Children's Hospital of Pittsburgh of UPMC. At examination under anesthesia (EUA), he showed microphthalmia and silvery opaque corneas. Due to the wide and indistinct limbal area, the horizontal corneal diameters were difficult to evaluate, but they appeared to be approximately 9.5 mm. Both corneas showed reticular vascularization and a grayish appearance with a central white opacity (Figure 1A,B). Despite the opaqueness of the cornea, it was possible to obtain a hazy view of the retina—which appeared flat, as subsequently confirmed with B‐scan (Figure 1C,D) and of the optic nerves. Digitally, the eyes were moderate to firm. High‐frequency ultrasound showed the absence of normal lenses and the presence of irido‐corneal adhesions. Anterior segment optical coherence tomography (AS‐OCT) highlighted the presence of some lens remnants adherent to the posterior corneal surface and vitreous coming up to the cornea, a clear sign of aphakia (Figure 1A,B). Axial length was 14.35 and 14.87 mm in the right eye (RE) and left eye (LE), respectively. Pachymetry was 420 and 401 μm, respectively. Extensive evaluations were carried out to exclude other congenital abnormalities or extra‐ocular conditions but resulted overall normal except for the presence of a slightly patent foramen ovale. Of note, ocular examination of both parents was unremarkable, and there was no family history of congenital ocular disease.

At 2 months, the patient was able to fix and follow light with both eyes; at 6 months, he started topical treatment for glaucoma control. Another EUA was performed when the child was 12 months old. The horizontal corneal diameters, though difficult to ascertain exactly, were 13 mm in either eye. Axial lengths were more increased than expected for age (20.70 mm in the RE, 20.86 mm in the LE). Both corneas were opaquer than previously and hindered the view of a proper red reflex. The pachymetry values were increased as well (1165 and 1174 μm in the RE and LE eye, respectively). AS‐OCT clarified that lens remnants became opaque. B‐scan showed the development of posterior staphyloma in both eyes. Bilateral Ahmed tubes were placed when he was 2 years old. At 6 years old, best spectacle‐corrected visual acuity was 20/1000 at 1 m away, with both eyes open and +28 diopter lenses in spectacles.

Given the findings of his examination and ultrasound, a panel of genes associated with anterior segment dysgenesis was performed in a CLIA‐certified laboratory, including the *FOXE3* gene associated with congenital primary aphakia. No *FOXE3* variants were detected on sequencing or array. An oligonucleotide array was also performed, and no possibly pathogenic genomic copy number variants were identified. Furthermore, it was confirmed that the three oligonucleotide probes within the *FOXE3* gene were present and provided further confirmation that the gene was intact.

Initially, whole exome sequencing was performed and identified three variants of unknown clinical significance, including: *JAG1* c.3346C>T (p.R1116W), paternally inherited; *RPGRIP1L* c.701C>T (p.T234I); and *PAX2* c.2T>A (p.Met1Lys), maternally inherited. The patient's ocular and systemic histories were not consistent with Alagille Syndrome (associated with *JAG1*) or COACH Syndrome, Joubert Syndrome, and Meckel Syndrome (associated with *RPGRIP1L*). Furthermore, a second variant or mutation was not identified in *RPGRIP1L*, which is necessary to cause the related symptoms as the associated conditions are inherited in an autosomal recessive manner. A repeat renal ultrasound and urine analysis were arranged to rule out Renal‐Coloboma Syndrome (associated with *PAX2*) and remained normal. The heterozygous missense variation, notated c.2T>C (p.Met1Lys), in *PAX2* may abolish the start codon, resulting in delayed translation. Polyphen‐2 and SIFT predict the *PAX2* variant to have a damaging effect, while MutationTaster predicts the variant to be a polymorphism with a loss of two amino acids, as the third amino acid of the PAX2 protein is also a methionine and the sequence may act as a replacement start codon [7, 8, 9]. Using the 2015 ACMG guidelines for the interpretation of sequence variants, the *PAX2* variant appears to be classified as a variant of unknown significance [10].

## 3 Methods

### 3.1 Human Studies and Subjects

This study was approved by the University of Utah Institutional Review Board (IRB) under IRB# 00131756 and the University of Pittsburgh under IRB STUDY 23060104. These studies conform to US Federal Policy for the Protection of Human Subjects. Experiments were undertaken with the understanding and informed written consent of both parents.

### 3.2 Ophthalmology

A retrospective review of the patient's medical records was performed.

### 3.3 Genetics

The patient's and his parents' DNA were collected by blood draw. Genomic DNA was extracted according to standard protocols. Novogene sequenced the whole genome using short reads (150 bp paired) to 30× depth coverage. Reads were mapped and variants called using GATK Best Practices (v 4.1) [11]. These variants were filtered using slivar (v 0.2.7) [12]. Variants were filtered out if there were (1) < 10 reads at that locus for each person in the trio, (2) present a population frequency > 0.1% in either the TOPMed (db151) and gnomAD (hg38 v3) databases, or (3) followed a dominant inheritance pattern [13, 14]. Variants were labeled de novo if the variant is present as heterozygous in the autosomal region in the child and absent in both parents. The variant was validated in a CLIA‐certified laboratory (Blueprint Genetics, Marlboro, MA, USA) using genomic DNA extracted from independent buccal samples. Prior to validation, the relative conservation of the affected residue was assessed using a multiple sequence alignment containing 120 mammal orthologs of the GJA8 protein [15]. A condensed set of 25 mammals was visualized using AliView [16]. Dates were obtained from TimeTree [17].

## 4 Conclusion and Results

**FIGURE 2:**
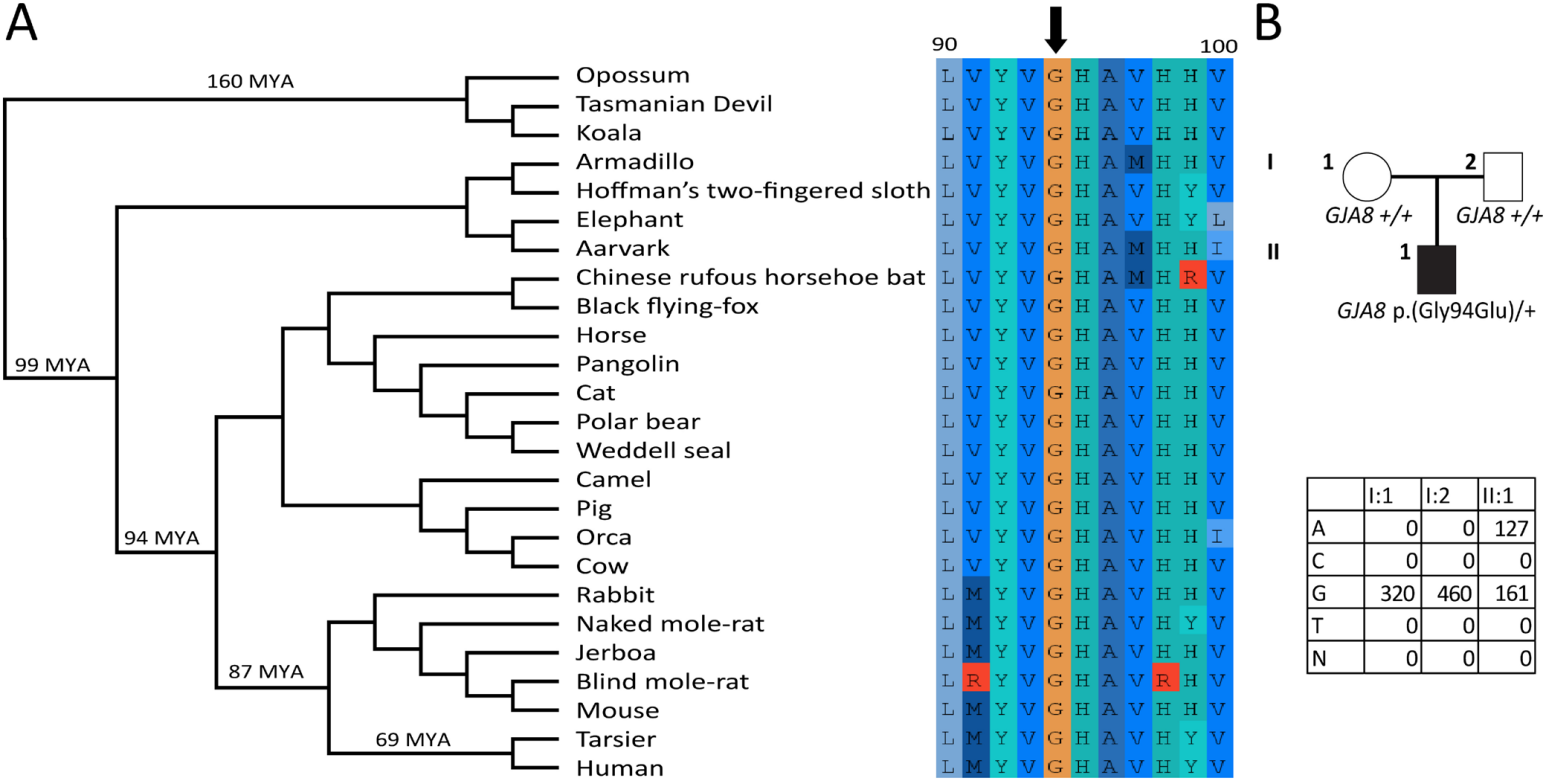
Conservation of G94 in GJA8 and validation results with pedigree. (A) A truncated multiple sequence alignment from the Hiller lab's 120 mammal genome alignment [15] using twenty‐five mammals spanning 160 million years (MYA). Phylogenetic tree indicates relatedness of mammals who's orthologous GJA8 sequence was used. Time on branches indicates approximately when lineage(s) diverged from humans. Color in multiple sequence alignment corresponds to relative amino acid chemistry. Numbers on top of alignment indicate amino acid position according to human reference. Arrow indicates glycine 94. (B) Pedigree and sequencing results of family at this locus. The lack of variation at residue 94 in GJA8 strongly suggests this residue is important for vision. The alteration of this conserved residue to another amino acid with drastically different size and biochemical properties made the variant a prime candidate to explain the patient's condition.

In light of these considerations, the genetic etiology of the patient's ocular condition remained unclear. Subsequent whole genome sequencing revealed that the heterozygous missense variant *GJA8* c.280G>A, p.(Gly94Arg) was the only *de novo* missense variant detected (Figure 2B). That variant was seen as a high priority because this gene has a known role in vision and the amino acid was revealed to be invariant in mammal orthologs (Figure 2A). The variant is predicted to be deleterious by all *in silico* tools utilized. In addition, the same amino acid change had been previously reported in a family with CA, although it was due to a different nucleotide mutation [6].

## 5 Discussion

CA is predominantly associated with *FOXE3* pathogenic variants, and this gene is therefore targeted for sequencing in patients showing this phenotype [1]. Other genes have been identified, often as responsible for CCO, whilst the absence of the lens is listed as a collateral finding and not as a causative factor for the corneal opacity itself. To date, the other identified genes include *HCCS* and *GJA8* [5, 6, 18, 19].

The *GJA8* (MIM *600897) gene encodes a transmembrane connexin protein (also known as connexin 50, or Cx50) that is necessary for lens growth and maturation of lens fiber cells. The encoded protein is a component of gap junction channels and functions in a calcium‐and pH‐dependent manner [6], which allows intracellular transport of ions, nutrients, and signaling molecules that are essential for proper lens growth and transparency. Lack of functional Cx50 can prevent proper fiber cell elongation and lens growth due to apoptosis or improper differentiation of lens fiber cells. A truncated or absent Cx50 protein disrupts developmental processes within the lens and leads to abnormal lens development. Effectively, pathogenic variants in *GJA8* have been identified to cause several types of autosomal dominant cataract. In some cases, cataract is associated with microcornea without any other systemic anomaly or dysmorphism [20]. Recessive inheritance of *GJA8*‐related disease has also been reported in a few consanguineous families [21, 22, 23]. Furthermore, the absence or malformation of the lens fiber cells can result in the failure of the lens to form properly, leading to CA. Expression of *GJA8* and the role of Cx50 in lens fiber cell elongation and differentiation are therefore key to understanding how such genetic defects impact the lens, including both cataract and aphakia phenotypes.

The *GJA8* c.280G>A, p.(Gly94Arg) missense variant identified in this study is absent in gnomAD, a large reference population database (*n* > 120,000 exomes and > 15,000 genomes) which aims to exclude individuals with severe pediatric disease [13]. The affected amino acid is highly conserved across mammals and other vertebrates, which suggests that this position may not tolerate variation (Figure 2A). A brief literature review reveals several independent individuals who possess anterior segment abnormalities at or near this variant, highlighting that individuals with this coding variant tend to present with aphakia, sclerocornea, and glaucoma (Table 1). For a more extensive review of documented variants in *GJA8*, see this review [25]. Consistent features of this *GJA8* variant include corneal opacity secondary to the absence of lens or the presence of lens residuals, microcornea, and bilateral glaucoma. Contrary to the previous study [6], we think that our patient did not have retinal coloboma but, in fact, developed a posterior staphyloma likely related to the raised intraocular pressure. In conclusion, this case report further confirms that *GJA8* should be tested in patients with CA who are negative for *FOXE3* or *HCCS* pathogenic variants.

**TABLE 1:** Review of variants in *GJA8* in patients with anterior segment abnormalities. Brief literature review revealed several independent cases of abnormalities which highlights the need to include *GJA8* in testing patients with anterior segment disorders.

*GJA8* variant	Diagnosis	Study
c.151G>A, p.(Asp51Ans)	Bilateral total corneal opacification and bilateral congenital cataracts from birth, whilst later developed bilateral glaucoma	[18] and possibly [19]
c.280G>C, p.(Gly94Arg)	Bilateral total corneal opacification, microcornea, bilateral glaucoma and left bupthalmos, bilateral rudimentary lenses, and no iridocorneal or kerato‐lenticular adhesions	[18] and possibly [19]
c.280G>C, p.(Gly94Arg)	Congenital aphakia, sclerocornea, microphthalmia, iris and optic disc coloboma, and bilateral primary glaucoma	[6]
c.280G>A, p.(Gly94Arg)	Microphthalmia, silvery opaque corneas, irido‐corneal adhesions, corneal vascularization, glaucoma, posterior staphyloma, increased ocular pressure, congenital aphakia	This paper
c.280G>A, p.(Gly94Arg)	Variant reported in large cohort with inherited eye diseases	[24]
c.281G>A, p.(Gly94Glu)	Microcornea, sclerocornea and cataractous disc‐like lenses, but no raised intraocular pressure	[18] and possibly [19]

## Author Contributions

**Sarah A. M. Lucas:** formal analysis, investigation, writing – original draft, writing – review and editing. **Elena Franco:** investigation, writing – original draft, writing – review and editing. **Hannah L. Scanga:** formal analysis, investigation, writing – review and editing. **Nathan L. Clark:** conceptualization, formal analysis, funding acquisition, writing – review and editing. **Ken K. Nischal:** conceptualization, formal analysis, funding acquisition, investigation, writing – review and editing.

## Ethics Statement

This study was approved by the University of Utah Institutional Review Board (IRB) under IRB# 00131756 and the University of Pittsburgh under IRB STUDY 23060104. These studies conform to US Federal Policy for the Protection of Human Subjects. Experiments were undertaken with the understanding and informed written consent of both parents.

## Consent

Written informed consent was obtained from the patient's parent for the publication of this case report and accompanying images.

## Conflicts of Interest

The authors declare no conflicts of interest.

## Data Availability

The data that support the findings of this study are available on request from the corresponding author. The data are not publicly available due to privacy or ethical restrictions.

## References

[1] J. Ernst , A. Medsinge , H. L. Scanga , et al., “Congenital Primary Aphakia,” Journal of American Association for Pediatric Ophthalmology and Strabismus 26, no. 1 (2022): 4.e1–4.e5, 10.1016/J.JAAPOS.2021.09.008. 35051625

[2] E. Franco , H. L. Scanga , and K. K. Nischal , “Variable Phenotype of Secondary Congenital Corneal Opacities Associated With Microphthalmia With Linear Skin Defects Syndrome,” American Journal of Medical Genetics Part A 191, no. 2 (2023): 586–591, 10.1002/AJMG.A.63043. 36369709

[3] L. M. Reis , E. A. Sorokina , L. Dudakova , et al., “Comprehensive Phenotypic and Functional Analysis of Dominant and Recessive FOXE3 Alleles in Ocular Developmental Disorders,” Human Molecular Genetics 30, no. 17 (2021): 1591–1606, 10.1093/HMG/DDAB142. 34046667 PMC8369840

[4] R. Happle , O. Daniels , and R. J. J. Koopman , “MIDAS Syndrome (Microphthalmia, Dermal Aplasia, and Sclerocornea): An X‐Linked Phenotype Distinct From Goltz Syndrome,” American Journal of Medical Genetics 47, no. 5 (1993): 710–713, 10.1002/AJMG.1320470525. 8267001

[5] C. J. Cape , G. W. Zaidman , A. D. Beck , and A. H. Kaufman , “Phenotypic Variation in Ophthalmic Manifestations of MIDAS Syndrome(Microphthalmia, Dermal Aplasia, and Sclerocornea),” Archives of Ophthalmology 122, no. 7 (2004): 1070–1074, 10.1001/ARCHOPHT.122.7.1070. 15249380

[6] F. Ceroni , D. Aguilera‐Garcia , N. Chassaing , et al., “New GJA8 Variants and Phenotypes Highlight Its Critical Role in a Broad Spectrum of Eye Anomalies,” Human Genetics 138, no. 8–9 (2018): 1027–1042, 10.1007/S00439-018-1875-2. 29464339

[7] I. A. Adzhubei , S. Schmidt , L. Peshkin , et al., “A Method and Server for Predicting Damaging Missense Mutations,” Nature Methods 7, no. 4 (2010): 248–249, 10.1038/nmeth0410-248. 20354512 PMC2855889

[8] P. C. Ng and S. Henikoff , “SIFT: Predicting Amino Acid Changes That Affect Protein Function,” Nucleic Acids Research 31, no. 13 (2003): 3812–3814, 10.1093/NAR/GKG509. 12824425 PMC168916

[9] J. M. Schwarz , D. N. Cooper , M. Schuelke , and D. Seelow , “MutationTaster2: Mutation Prediction for the Deep‐Sequencing Age,” Nature Methods 11, no. 4 (2014): 361–362, 10.1038/nmeth.2890. 24681721

[10] S. Richards , N. Aziz , S. Bale , et al., “Standards and Guidelines for the Interpretation of Sequence Variants: A Joint Consensus Recommendation of the American College of Medical Genetics and Genomics and the Association for Molecular Pathology,” Genetics in Medicine 17, no. 5 (2015): 405–423, 10.1038/gim.2015.30. 25741868 PMC4544753

[11] G. A. van der Auwera , M. O. Carneiro , C. Hartl , et al., “From FastQ Data to High‐Confidence Variant Calls: The Genome Analysis Toolkit Best Practices Pipeline,” Current Protocols in Bioinformatics 43, no. 1 (2013): 11, 10.1002/0471250953.BI1110S43. PMC4243306 25431634

[12] B. S. Pedersen , J. M. Brown , H. Dashnow , et al., “Effective Variant Filtering and Expected Candidate Variant Yield in Studies of Rare Human Disease,” NPJ Genomic Medicine 6, no. 1 (2021): 1–8, 10.1038/s41525-021-00227-3. 34267211 PMC8282602

[13] K. J. Karczewski , L. C. Francioli , G. Tiao , et al., “The Mutational Constraint Spectrum Quantified From Variation in 141,456 Humans,” Nature 581, no. 7809 (2020): 434–443, 10.1038/s41586-020-2308-7. 32461654 PMC7334197

[14] S. T. Sherry , M. Ward , and K. Sirotkin , “dbSNP—Database for Single Nucleotide Polymorphisms and Other Classes of Minor Genetic Variation,” Genome Research 9, no. 8 (1999): 677–679, 10.1101/GR.9.8.677. 10447503

[15] N. Hecker and M. Hiller , “A Genome Alignment of 120 Mammals Highlights Ultraconserved Element Variability and Placenta‐Associated Enhancers,” GigaScience 9 (2020): 1–10, 10.1093/gigascience/giz159. PMC6941714 31899510

[16] A. Larsson , “AliView: A Fast and Lightweight Alignment Viewer and Editor for Large Datasets,” Bioinformatics 30, no. 22 (2014): 3276–3278, 10.1093/bioinformatics/btu531. 25095880 PMC4221126

[17] S. Kumar , G. Stecher , M. Suleski , and S. B. Hedges , “TimeTree: A Resource for Timelines, Timetrees, and Divergence Times,” Molecular Biology and Evolution 34, no. 7 (2017): 1812–1819, 10.1093/molbev/msx116. 28387841

[18] A. S. Ma , J. R. Grigg , I. Prokudin , M. Flaherty , B. Bennetts , and R. V. Jamieson , “New Mutations in GJA8 Expand the Phenotype to Include Total Sclerocornea,” Clinical Genetics 93, no. 1 (2018): 155–159, 10.1111/CGE.13045. 28455998

[19] A. Ma , S. Yousoof , J. R. Grigg , et al., “Revealing Hidden Genetic Diagnoses in the Ocular Anterior Segment Disorders,” Genetics in Medicine 22, no. 10 (2020): 1623–1632, 10.1038/S41436-020-0854-X. 32499604 PMC7521990

[20] N. Quiroz‐Casian , O. F. Chacon‐Camacho , T. Barragan‐Arevalo , et al., “Sclerocornea‐Microphthalmia‐Aphakia Complex: Description of Two Additional Cases Associated With Novel foxe3 Mutations and Review of the Literature,” Cornea 37, no. 9 (2018): 1178–1181, 10.1097/ICO.0000000000001655. 29878917

[21] R. L. Gillespie , J. O'Sullivan , J. Ashworth , et al., “Personalized Diagnosis and Management of Congenital Cataract by Next‐Generation Sequencing,” Ophthalmology 121, no. 11 (2014): 2124–2137, 10.1016/J.OPHTHA.2014.06.006. 25148791

[22] S. P. G. Ponnam , K. Ramesha , S. Tejwani , B. Ramamurthy , and C. Kannabiran , “Mutation of the Gap Junction Protein Alpha 8 (GJA8) Gene Causes Autosomal Recessive Cataract,” Case Reports 2009 (2009): bcr0620091995, 10.1136/BCR.06.2009.1995. PMC3029104 21720542

[23] W. Schmidt , N. Klopp , T. Illig , and J. Graw , “A Novel GJA8 Mutation Causing a Recessive Triangular Cataract,” Molecular Vision 14 (2008): 851–856. 18483562 PMC2375854

[24] W. Li , N. Qu , J. K. Li , et al., “Evaluation of the Genetic Variation Spectrum Related to Corneal Dystrophy in a Large Cohort,” Frontiers in Cell and Development Biology 9 (2021): 632946, 10.3389/FCELL.2021.632946. PMC8012530 33816482

[25] J. L. Jones and K. P. Burdon , “Evaluating Gap Junction Variants for a Role in Pediatric Cataract: An Overview of the Genetic Landscape and Clinical Classification of Variants in the GJA3 and GJA8 Genes,” Expert Review of Ophthalmology 18, no. 1 (2023): 71–95, 10.1080/17469899.2023.2160320.

